# Bacteriophage Based Biosensors: Trends, Outcomes and Challenges

**DOI:** 10.3390/nano10030501

**Published:** 2020-03-11

**Authors:** Zahra Aliakbar Ahovan, Ali Hashemi, Laura Maria De Plano, Mazaher Gholipourmalekabadi, Alexander Seifalian

**Affiliations:** 1 Department of Microbiology, School of Medicine, Shahid Beheshti University of Medical Sciences, Tehran 1985717443, Iran; zahraahovan@sbmu.ac.ir; 2 Department of Chemical, Biological, Pharmaceutical and Environmental Sciences, University of Messina, 98166 Messina, Italy; ldeplano@unime.it; 3 Cellular and Molecular Research Centre, Iran University of Medical Sciences, Tehran 1449614535, Iran; 4 Department of Tissue Engineering & Regenerative Medicine, Faculty of Advanced Technologies in Medicine, Iran University of Medical Sciences, Tehran 1449614535, Iran; 5 Nanotechnology & Regenerative Medicine Commercialization Centre (NanoRegMed Ltd.), London BioScience Innovation Centre, London NW1 0NH, UK

**Keywords:** bacteriophage, biosensors, nanomaterials, foodborne pathogens, bacteria, smart materials, nanotechnology, nanoparticles, coronavirus

## Abstract

Foodborne pathogens are one of the main concerns in public health, which can have a serious impact on community health and health care systems. Contamination of foods by bacterial pathogens (such as *Staphylococcus aureus, Streptococci*, *Legionella pneumophila*, *Escherichia coli*, *Campylobacter jejuni and Salmonella typhimurium)* results in human infection. A typical example is the current issue with Coronavirus, which has the potential for foodborne transmission and ruling out such concerns is often difficult. Although, the possible dissemination of such viruses via the food chain has been raised. Standard bacterial detection methods require several hours or even days to obtain the results, and the delay may result in food poisoning to eventuate. Conventional biochemical and microbiological tests are expensive, complex, time-consuming and not always reliable. Therefore, there are urgent demands to develop simple, cheap, quick, sensitive, specific and reliable tests for the detection of these pathogens in foods. Recent advances in smart materials, nanomaterials and biomolecular modeling have been a quantum leap in the development of biosensors in overcoming the limitations of a conventional standard laboratory assay. This research aimed to critically review bacteriophage-based biosensors, used for the detection of foodborne pathogens, as well as their trends, outcomes and challenges are discussed. The future perspective in the use of simple and cheap biosensors is in the development of lab-on-chips, and its availability in every household to test the quality of their food.

## 1. Introduction

Over the last several decades, the prevalence of food poisoning has become a major world health issue. This may be due to the increase in intercontinental transportation of food. Dr. Oliver, at the University of Tennessee, TN, USA, an expert in the foodborne pathogens, has recently reported these problems in detail [1]. The Centers for Disease Control and Prevention (CDC), Atlanta, GA, USA assesses more than 36 million cases of the disease annually, are due to foodborne and waterborne pathogens infections [2]. The most common causes of food poisoning are diarrhea, nausea, vomiting and stomach cramps. Food poisoning is dangerous for the elderly, as well as children, patients with chronic health conditions or weakened immune systems [3,4].

## 2. Common Foodborne Pathogens

According to the World Health Organization (WHO, Geneva, Switzerland, 2015, www.who.int), there are many possible causes of food poisoning, including; bacteria, viruses, poison, parasites, and many others. Microorganisms involved in food poisoning are generally present in all kinds of foods, especially in fruits, vegetables, as well as supermarket ready-made foods and takeaways [5]. The main pathogens responsible for serious foodborne disease outbreaks are *Listeria monocytogenes, Staphylococcus aureus*, *Salmonella*, *Campylobacter*, *Cryptosporidium* and *E. coli* 0157:H7 [6].

## 3. Types of Common Methods Used for the Detection of Pathogens

Conventional methods for the detection of foodborne pathogens depend on specific biochemical and microbiological tests [7]. These methods are time-consuming depending on the time it takes to pre-enrichment of the microorganisms and then culturing them on selective media. These methods are time-consuming, depending on the time it takes for the pre-enrichment of microorganisms and then culturing them on selective media. 

In particular, the major problems of current standard technologies are their enrichment steps and time-consuming up to 7–10 days, resulting in inconvenience in many industrial applications, particularly in food [8]. Moreover, viable bacterial strains in the environment can become non-cultivable (viable-but non-cultivable (VBNC)) leading to an underestimation of pathogen numbers or a failure to isolate a pathogen from a contaminated sample. Successively, mass spectrometry has been proposed to increase the speed and sensitivity of culture methods, but these methods have high a cost and require expertise for analyze and interpretation of the data. On the contrary, biochemical immunoassays, such as ELISA, although simple and rapid, can have low sensitivity for the detection of pathogens.

**Figure 1:**
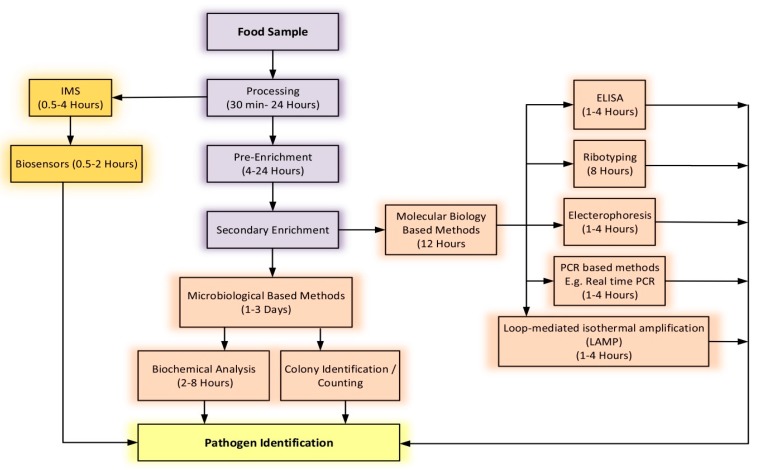
The steps involved in the diagnosis and analysis of food samples using common techniques and the time it takes to detect the pathogen.

Several different types of nucleic-acid-based assays have been developed and used as a faster technique to detect foodborne pathogens, for example, amplification (PCR), microarrays and biochips [9]. However, PCR techniques of detection, as well as recent multiplex-PCR and reverse transcriptase PCR (RT-PCR) are inefficient to analyze large sample volume without pre-enrichment and have high costs that renders them difficult for regular use [10]. Figure 1 shows the steps involved in the diagnosis and analysis of food samples, using current standard techniques, and the time it takes to detect the pathogen.

Consequently, recently, many researchers in the multidisciplinary team been working in research and development (R and D) of a biosensor, with the following specification, fast report output, simple, specific and sensitive devices able to in-situ, real-time monitoring, at low cost. There have been a number of emerging biosensors technologies, show potential approaches for in situ analysis of pathogen detection. This research aimed, critically review recent advances in biosensors that use bacteriophages or phage-derived as bio-probes for food pathogen detection.

## 4. Biosensors in Foodborne Pathogen Detection

**Figure 2:**
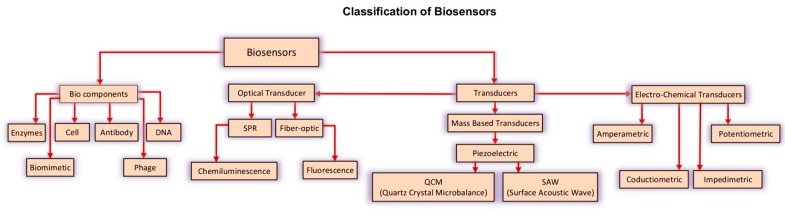
Schematic representation of the components involved in a biosensor devise.

The Biosensors are simple and rapid devices, based on organic probes, which are able to identify biological analytes, such as microorganisms, viruses, and biomolecules [11]. The biosensor is commonly composed of a biologically active sensitive element (biological element) and an electronic part (sensor or transducer). The operating principles are as follows: “the biological element” interacts with the substrate to be analyzed and a transduction system; “the sensor” converts the biochemical response into an electrical signal. This signal digitized into a numeric value, giving the final information. Biosensors can be classified according to the transduction technologies used. In the past decade, different groups of transductions have been introduced; these have led to the formation of three main classes: Optical, mass-based and electrochemical transducers (Figure 2) [12]. The front part of the biosensor, the probes, plays a major role in the identification and detection of the pathogens. These give biosensors the ability to analyze a wide range of complex samples in various fields, including the diagnosis of food pathogens, clinical diagnosis and environmental monitoring. Biosensors have been used and played as a useful tool for the direct detection of the pathogen in the factories during the food processed food. Unlike microbiological and molecular methods, biosensors can detect the pathogen immediately and accurately, and this helps to detect the contamination level and the type of food contamination [6].

## 5. Bio-Probe

**Figure 3:**
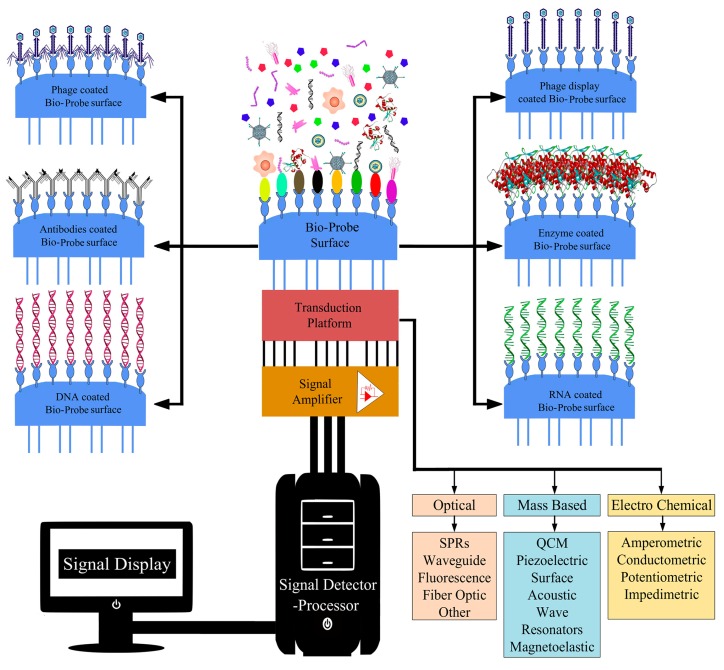
The schematic diagram shows the types of bio-probes and transducer in the biosensor. The figure is shown how the device works to detect pathogens.

Bio-probes are the most important components of biosensors because responsible to bind and identify the analytes targets. The two main characteristics of bio-probes are specificity and high affinity for the analyte. Figure 3 shows the different components of a diagnostic sensor. The common bio-probe, used in biosensor devices, are antibodies, proteins, DNA/RNA aptamers and carbohydrates. However, many of these are usually susceptible to environmental conditions and the need for laborious immobilization methods for binding on the sensor substrate. Only recent studies employed bacteriophages (or phage) or derived-phage, such as phage receptor binding proteins (RBPs) and most lately phage-display peptides (PDPs) like a valid alternative of standard bio-probes.

### 5.1. Phages Wild Type

**Figure 4:**
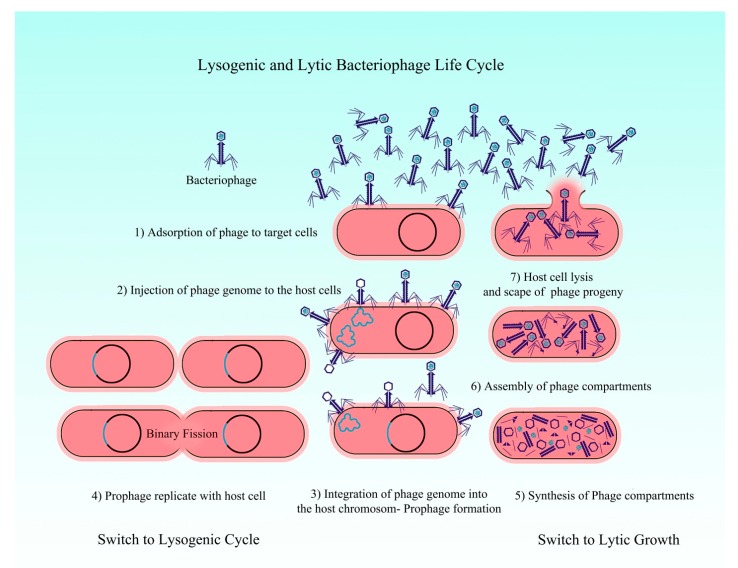
Conversion of lysogenic and lytic state of virulent state in phage-infected bacteria.

Bacteriophages (phages) are viruses ubiquitous in all environments, including soil, food ground and surface water. They specifically bind the host bacteria and inject their DNA to begin the multiplication and propagation of mature virions [13]. Phages can propagate new virions in two ways: Lytic or lysogenic cycle (Figure 4). 

Lytic cycle: Phages attach to their host bacteria, insert their DNA and take over the host machinery to dissemination new virions that lyse the bacteria and infect a new host (lytic phages). The host cells provide molecular blocks and enzymes that are needed to multiply the phage genomes and generate a progeny phage. Phages produce several endolysins and holin lysis proteins inside the host cell. Holins are small proteins in the cytoplasmic membrane of host bacteria that lead to peptidoglycan cell wall bacteria lysis by endolysins and release of produced phages. These phages in the external environment can infect and destroy other adjacent bacteria. In a wide range of bacterial species, lytic phage produce infection specifically [14].

Lysogenic cycle: Integrate their genome into the host DNA, remain latent until they arouse for replication and dissemination (lysogenic phage). A Prophage, contained in the host cell, is called lysogen. While, this DNA in the host genome is called a prophage, the lysogenic cycle can continue indefinitely, except in some cases, such as adverse conditions of the environment and bacteria exposure to stress [14].

Consequently, the bacteriophage used like bio-probe in biosensor devises offer several advantages, such as (1) specificity to host bacteria, consequently efficient bacteria screening [15], (2) easy to generate mass quantities of progeny phages, due to their short replication time, (3) ability to tolerate critical conditions, such as organic solvents and large range of pH and temperature [16]. It is important to understand the characteristics of phages, such as the physical and chemical properties, in order to design the biosensor platform able to bind the phage without losing their ability to recognize the bacteria target. Phages can bind to the gold surface trough van der Waals bonding, hydrophobic bonding and covalent bonding between the gold surface and the amine and thiol groups. This strategy is also used in surface plasmon resonance (SPR) sensor and quartz crystal microbalance (QCM) sensor using phage as a probe for pathogen detection [17]. Nevertheless, optimal condition of physical absorption has been found for lytic phages able to detect *S. aureus* by SPR with detection limit ~10^4^ cfu/mL^−1^, or using magnetic elastic sensors [18] capable of detecting *Salmonella* (detection limit ~10^3^ cfu/mL^−1^) in milk and fat-free [19]. However, physical absorption has several limitations, such as non-specific and weak bonding between phage and sensor surface, leading to desorption during the analytic detection and low surface coverage of deposited phages [17].

However, the chemical bond gives greater stability to the system. One of the main factors in these methods is the creation of a strong chemical bond between the phages and biosensor surfaces, which leads to the development of a stable capture system. In this case, the purity of the suspension of phages, such as their chemical properties, are important to be aware of before performing any chemical reactions [20]. Gervais et al. developed an oriented immobilization of T4 phages using the commonly recognized streptavidin-biotin reaction. The tail-phage was able to detect *E. coli* through the expression of biotin in the T4 head, and using the streptavidin-coated gold surface [21]. Moreover, the T4 phage has been used in the functionalization of magnetic nanobeads, and is used to capture and concentrate E. *coli* from milk by Mortari et al. The successive lysis of the bacteria-binding phage leads to the analysis of endoplasmic material through impedance, with the potential detection limit of 10 CFU/chamber in 30 min [22].

However, the size of the phage particles can be a limitation in any biosensor surfaces, such as in nanoscale devises. The phages have an enzyme activity relative to their bacterial host [23]. This kind of enzymatic activity on the biosensor surface causes contradictory signals that contribute to the efficiency of the pathogen diagnosis. Moreover, results recommend that whole phage bound on sensor platforms miss their bacterial binding capacity upon drying [24]. As the phages fall on the biosensor surface after upon drying, it likely makes their tail fibers unable to bind to their bacterial host. In order to overcome these limitations, engineered phages or derived phages have been applied in the development of biosensors devises, as discussed later in this review article.

### 5.2. Engineered Phages

With novel advances in the field of genetic engineering, new phage probes have been designed to increase the capability or to overcome the limits of the wild-type phages. Recently, N. Wisuthiphaet and co-workers used engineered bacteriophage, phage T7-ALP, which expresses alkaline phosphatase to detect *E. coli* in beverage samples. After infection of the host bacteria, the overexpression of alkaline phosphatase provides, after the mission of the substrate ELF-97, fluorescence imaging results in high-detection sensitivity of 100 bacteria for gram in 6 h [25]. In these cases, the specificity of phages, combined with engineered techniques, have permitted the easy and rapid identification of the target.

Reporter Phages - A reporter phage is an engineered phage to produce a signal upon infection of target bacteria. This group of phages detects only live and functional cells because when the phages infect the target bacteria, they activate the cells’ machinery to produce a readily detectable signal, which indicates that the target is both present and viable. This technique is an advanced method of bacteria typing [8]. Phage lambda-based cloning vectors bearing a functional bacterial bioluminescence lux operon was used to improve the reporter phage method. Here, lux operon is expressed in the process of infection by phage in the host cell as part of the phage gene [13]. ‘‘Lux-’’ or ‘‘Gfp-’’ expressing phages were successfully used in *Salmonella enterica* and pathogenic *E. coli* O157:H7 detection, as well as for detection of *Listeria* and *Mycobacteria* [26]. In a study by Zhang et al., the design of a phage that contains luciferase NanoLuc (Nluc), reporter phage, was able to detect *E. coli* 0157:H7, about 5 CFU/mL, in a food sample by bioluminescence over 9 h [27]. The benefit is that the phage specificity and the strength of the analysis of the phage remove the essential for purification or lengthy sample preparation [28]. Moreover, recently has been shown a proof-of-concept drinking water diagnostic assay for low-cost, rapid and sensitive detection of *E. coli* using T7 reporter phage. T7 was engineered to express the luceriferase, NanoLuc (NLuc), after the fusion of crystalline cellulose. This novel chimeric reporter allow the detection of <10 CFU mL^−1^ *E. coli* in 3 h from 100 mL water sample [29]. However, some structural disadvantages associated with this group of phages also have to be stated. To study the structure of these phages there is a need to have accurate genetic information as their structure is labor-intensive. The capacity of the phage capsid naturally creates limitations for the amount of genetic material that can be presented in the phage genome. General ways to exhibit reporter genes contain recombination, transposition, direct cloning and homologous [8].

### 5.3. Phage Display Peptides

The phage display technology must be considered separately. Since, it was first reported in 1985 in Science, phage display technology has evolved as a powerful method for discovery of antigen-specific peptides. A researcher found that bacteriophages can be genetically manipulated through the incorporation of exogenous (poly) peptides into its coat proteins, making a peptides phage library. The filamentous phages, such as fd and M13, are the most commonly used vector to create random peptide display libraries. Filamentous phages appear as a flexible rod-shaped structure, with a total length of 880 nm and a diameter of 6.6 nm. Its circular genome, of 6000–8000 bases, is enclosed in a coat composed of up of 2700 copies of major coat protein (pVIII), 3–5 copies each of the minor proteins (pIII, pVI and pVII, pIX), on the two ends of the phage particle. In particular, the foreign peptides (length from 6 to 21 amino acids) have been displayed fused to surface exposed N-terminus of all coat proteins. However, the most commonly-used coat proteins for phage display are pIII and pVIII. The phage display libraries consisted of up to 10^10^ different variants of phage particles with the linear random peptides [30]. This allows ligands to be screened that have a high affinity towards the desired antigen, such as eucariotic cells, bacteria or inorganic material. The phage clones were found to bind, with high affinity, the target, which can be used as a bio-probes in biosensor devises. In this way, for all possible targets, it is possible to find a “phage-clone” able to recognize them. The advantage are robust, stable and resistant to temperature variations and hard pH conditions, such as the wild-type. Moreover, the phage clone presents several copies of peptide of interest, thus increasing the avidity of the specific target binding, compared to the classic wild-type [31]. It has introduced several opportunities in various fields, including vaccine design, targeted drug delivery and antiviral studies [31]. These characteristics make phages an attractive choice as probes for developing biosensors in several fields [32,33].

### 5.4. Phage Receptor Binding Proteins

Recently bacteriophage receptor binding proteins (RBPs) have been developed into tools that make use of their high specificity [34]. RBPs offer several advantages compared to other elements for example antibodies, including ligand specificity, greater stability, and affinity even against carbohydrate epitopes [35]. The rapid detection of pathogens prevents disease progression and spread. RBPs are a practical technology for bacterial diagnosis.

The phage receptor proteins determine the phage-host characteristics and are also considered as suitable as biological agents. One of the advantages of the RBP is that, without lysis, the bacterial components of the bacterial cell proliferate through the agglutination and release of the DNA of the pathogen. RBP often has good resistance to environmental factors, such as temperature and pH [36]. Poshtiban and coworkers anchored the phage NCTC12673 presenting RBP protein Gp047 on magnetic beads. The modified seeds were used to extract Campylobacter from milk and chicken samples. In samples infected with 10^2^ CFU/mL of Campylobacter cells through RT-PCR, it was a prominent improvement measure of more than 80% in 3 h. To confirm the specific adsorption of phage to Campylobacter, *S. typhimurium* was used as a negative control [37]. More effort is needed to reach commercial biosensors, given that the initial experimental results indicate that RBPs are capable of being used as diagnostic agents in diagnosing pathogens.

## 6. Phage-Based Biosensors

In recent years, biosensors have been developed as new diagnostic methods to minimize the limitations of common pathogen detection methods. In phage-based biosensors, bacteriophage is attached to the sensor surface, and consequently, it can detect the pathogen in the sample [38]. The main advantages of this method are sensitive, accuracy and reliably. Bacteriophage-based biosensors have been used for direct diagnosis of pathogens in fresh foods such as milk [19,39], and water [40] food matrices [41]. The Table 1 summarizes the advantages of biosensors in pathogenesis.

### 6.1. Phage-Based Optical Biosensors

Optical-based bioassay systems are used for rapid diagnosis of pathogens in different experimental conditions, with high sensitivity and compatibility. Optical biosensors are used as more suitable diagnostic systems for the detection of pathogens. Detection in optical biosensors is based on the variations induced in the light properties, such as refractive index, wavelength and polarization [42]. Currently, BIACORE 3000 biosensor and SPREETA biosensor as commercial optical biosensors are used for the detection of foodborne pathogens. BIACORE 3000 biosensor is used for detecting *L. monocytogenes*, with sensitivity of 1 × 10^5^ cells/mL in milk. *Salmonella enteritidis* and *E. coli* O157:H7, and *S. typhimurium* and *S. enteritidis*, can be successfully detected by BIACORE 3000. and SPREETA biosensors, respectively [43]. Wavelengths-based biosensors enable real-time monitoring of biomolecular interactions by evaluating the kinetics and affinity of the interactions [42]. Planar optical waveguides contain an optically transparent guiding layer with a refractive index, which is higher than the substrate layers. The optical waveguide geometry provides an excellent surface for functionalization and pattering of different recognition elements, and enable the simultaneous detection of multiple analytes in a single waveguide transducer [44]. Optical techniques are separated into two major subclasses, fluorescence and label-free, based on their working platform. The most used technique for these biosensors is the measure of the changed fluorescence, in absorbance or luminescence, of the biosensor surface after analyte recognition. Furthermore, one of the advantages of the optical biosensor design of label-free biosensors is the detection of a specific and susceptible bacterial pathogens [45]. The most employed techniques for bacterial detection are fluorescence/phosphorescence spectrometry, surface Plasmon resonance (SPR), and bio/chemiluminescence.

#### 6.1.1. Surface Plasmon Resonance Sensors (SPR)

For the first time in 1990, SPR was used to detect a spectrum of materials [46]. SPR biosensors are optical sensors that use special surface plasmon-polaritons—electromagnetic waves, to monitor the interactions between an analyte in solution and a recognition layer, such as recognize molecules and phages, immobilized on the SPR sensor surface. SPR biosensing, as a spectroscopic technique, enable the quantitative and real-time detection of the binding events without labeling the interacting molecules. The optical system is comprised of an optical surface, a light emitting diode (LED), a glass prism and a photodiode array. The molecular interactions at the surface cause changes in the refractive index, leading to changes in the SPR angle of the reflected light. The photodiode array detects SPR angle changes, and expresses the signals as a response unit (RU). RU is directly proportional to the total mass of the bound ligands. The chips in these sensors usually contains a gold surface functionalized with specific biorecognition elements by chemical bounds [47]. Advanced SPR biosensors have been designed to detect pathogens using a variety of bio-probes in diagnosis of pathogens such as antibodies [48,49], bacteriophages [50] and lectins [51]. Bacteriophages are designed as diagnostic probes for specific detection of pathogens at the SPR level. For example, Singh et al. used immobilized engineered tail spike proteins derived from the P22 bacteriophage onto gold surfaces using thiol-chemistry, in order to selective real-time analytical detection of *Salmonella* with the sensitivity of 10^3^ CFU/mL^−1^ [24]. This technique successfully detects *E. coli* O157:*H7*, methicillin-resistant *S. aureus* (MRSA) [52], *S. aureus, E. coli K12* [13] and hepatitis B virus (HBV) [53]. Full-length recombinant Det7 phage tail proteins (Det7T) are recently used in novel SPR devise for detection of *Salmonella enterica serovar typhimurium*. Det7T is covalently immobilized on gold-coated surfaces by amine-coupling, and can specifically bind to *S. typhimurium*. Rapid detection (~20 min) of 5 × 104^−5^ CFU/mL *S. typhimurium* in water and 10% apple juice was observed by this biosensor [54]. In Table 2 several microorganisms that have been detected using this technique are summarized. The Plasmon waveguide resonance (PWR) spectroscopy, as a relatively new biophysical method, has the structures, which can couple high sensitivity of the SPR sensors and the small resonance width of the dielectric WG sensors. The PWR consists a glass substrate, a thin metallic layer, and a dielectric layer on the top of the metallic layer. The metallic layer plays a very important role in exciting the dielectric waveguide modes (transverse magnetic (TM), and transverse electric (TE). As an example, optical metal clad leaky waveguide (MCLW) sensor can detect Bacillus subtilis var. niger using index changes, scattering and fluorescence from bacterial spores bond to immobilized antibody [55].

#### 6.1.2. Bioluminescence Sensors

Bioluminescence through the oxidation of organic compounds (Luciferin), due to the enzyme Luciferase, produces visible light in the living organisms. Commonly in marine environments, some bacteria, including Vibrio strains, are widely and abundantly used as luminescent organisms. The ATP Bioluminescence tests are a sensitive, fast and simple ways for bacterial detection. In this method, the bacterial cell is lysed and releases intracellular ATP which is measured by luciferase bioluminescence reaction. The problem of this method is the low specificity in both Adenylate kinase (AK) and ATP diagnosis. Wu et al. showed that the rate of the release of AK from the bacterial cell depends on the growth stage, phage type infection time and type of bacteria [63].

#### 6.1.3. Fluorescent Bioassay

In this way, the fluorescent combination with bacteriophage plays a role in the diagnosis of pathogens. In this method, fluorescent blended bacteriophages are involved in detecting and binding to the host bacteria. Phage-bacteria is discovered using flow cytometry or epifluorescent filter technique. The average sensitivity reported so far is around 10^4^ CFU/mL^−1^ for flow cytometric and around 10^2^–10^3^ CFU/mL^−1^ for epifluorescent microscopy detection [67,68]. Goodridge et al. combined this method with immunomagnetic separation. In this way they could be able to detect 10 to 10^2^ CFU/mL^−1^ *E. coli* O157: H7 in synthetic milk after an enrichment [39] phase for 10 h and 10^4^ CFU/mL^−1^ of *E.coli* O157:H7 in broth [69]. This method is also used to identify bacterial toxins [70]. Goldman et al. displayed a practical phage to select a 12-per peptide that could bind to staphylococcal enterotoxin B (SEB), which cause food toxication. They could detect low 1.4 ng of SEB per sample in a fluorescence-based immunoassay using a labeled SEB-binding phage [70].

### 6.2. Electrochemical Biosensors

#### 6.2.1. Amperometric Biosensors

Amperometric biosensors are based on measuring the flow generated by oxidation or reduction in response to analyte bio-receptor reactions. In this case, a bio-receptor is usually an enzyme, such as glucose oxidase, horseradish peroxidase (HRP) and alkaline phosphatase (AP) [71]. In general, this technology includes a thin plate of gold or platinum or carbon. The main advantage of these biosensors is that they are simple and easy to use, and at the same time, highly sensitive. The limitation of this method is low specificity due to interference with active inhibitors, and then the signal inaccuracies. In this method, a quantification of coliform *E. coli* K-12 with intermediary phages and intracellular release of bacterial enzymes, such as D-galactosidase and carbon oxides can be investigated [72]. This sensor detected 1 CFU/100 mL of bacteria from the sample, but need to pre-incubation phase. Neufeld et al. combined the phage typing technique with amperometric for the specific detection of *Mycobacterium smegmatis, E. coli K12*, and *Bacillus cereus* [72]. The basis of these sensors is that through phage infection, it causes bacterial leakage and the spread of intracellular bacterial content, including the enzyme. Enzymatic activity is measurable in a specific substrate. The recent advanced reports, nanoparticle transducers were used to reduce the limitation of electrochemical biosensors. The Gold nanoparticle is one of the most common nanoparticles used in MRI, biosensors and targeting drug delivery for treating brain diseases, significantly increased electrodes sensitivity to the detection of pathogens. Xu et al. designed micro-gold electrodes with phage T4 for the detection of *E. coli*. The sensitivity of this biosensor is in the range of 1.9 × 10^1^–1.9 × 10^8^ CFU/mL bacteria [17]. Table 3 are summarized in several nanoparticles that have been used for detecting microorganisms.

#### 6.2.2. Electrochemical Impedance Spectroscopy (EIS) Biosensors

Electrochemical impedance spectroscopy (EIS) is a powerful technique in detecting the electrochemical system. Functional sinusoidal in the system measures the changes in the electrical impedance in the medium. The analysis is carried out based on changes in capacitance, conductance, and impedance. The capacity is often reduced due to the process of microbial metabolism and impedance decreases [79]. Here, bacteriophages are used as diagnostic probes to detect pathogens at the electrode surface. In a study, the impedance was reduced as bacterial concentration enhanced, which is contrary to normal attachment of entire cells on EIS, a promising electrochemical biosensor. Bacteria can be detected by capturing electrons on the electrode surface. In EIS electrochemical sensors with bacteriophage, as a probe, are used to detect bacteria, trough catching the bacteria target the immobilized phages on electrodes, functional groups [17,80]. The main cause of this is the activity of lytic phages on bacteria, which causes the intracellular content to drop out and decrease the conductivity. *E. coli* was easily detected in pure culture media or inoculated samples in a range between 10^4^–10^7^ CFU mL^−1^ using this method [81]. Webster et al. extend an impedimetric microelectrode array biosensor bacteriophage-based for the detection of bacteria. The results have shown that decreased the width and gap of an electrode and using the working solution with lower relative dielectric permittivity can enhance the sensitivity of impedimetric biosensors for pathogenic bacteria [82]. Graphene is wonder materials with superior properties and using graphene a screen-printed graphene electrode (SPE) for the detection of *S. arlettae*. Specific lytic phage against S. arlettae was immobilized on the sensor surface for quantitative analysis of the bacterial cells and capturing bacteria using EIS biosensor. Accordingly, the increase in the concentration of bacteria (2–2 × 10^6^ CFU/mL) leads to an increased quantity of charge transfer resistance (R_ct_). The limit of detection was defined as around 2 CFU/mL [83].

## 7. Benefits and Challenges

In these techniques, phages are widely used as bio-probes to detect pathogens. Phages are specific for host bacteria and have different characteristics, including that they are easy to amplify and cheap to produce, are resistant to temperature and pH-degradation, as well as organic solvents [74,75,76,77]. Moreover, cloning of engineered phage, derived from phage display libraries, make it able to develop new phages with on demand binding unites on their surfaces. This makes the phages a very promising candidate for biosensor applications [13]. Optimization of phage size, expression of binding units on phage’s surface for specific binding to bacteria, are of the main troubleshooting in development of new phage-based biosensors. 

## 8. Conclusions and Remarks

Biosensors have already demonstrated huge potential in many fields, including detection of viruses and diseases in patients, as well as in the identification of food pathogens. Many of these devices are still at laboratories’ experimental bases and translation to the commercial product has been slow. The main problem, includes a signal-to-noise ratio, caused by the separation of signals from bacteria, due to the unwanted signal “noise” from the samples. Therefore, sensitivity and repeatability are the major problems in the biosensors. High specificity is one of the main features of biosensors in diagnosis assisted by sensitivity, without spending time for the pre-enrichment phase.

Here, a summary of bacteriophage-based bio-probes and their protein receptors are discussed. At the same specificity of the antibodies and nucleic acid, the phage probes are strong, resistant and cost-effective. Recent efforts have led to advances in methods in which phage, based on its chemical properties, is positioned on the surface of the sensor and provides a consistent and stable surface that results in a widespread diagnosis. Although, phage-based and RBP-based systems have improved food quality control, it is still an emerging system. Significant advances have been made in this area and there is a clear and promising future.

Finally advances in cutting edge R and D on nanomaterials and smart materials, including recent work on graphene-based biosensors [84,85], will speed-up development and translate to commercialization. The current situation with Coronavirus is a warning to all on how important of fast and reliable detection of viruses in reducing mortality.

## Figures and Tables

**Table 1:** Ideal properties incorporated for microbial biosensor.

**Assay time**	Near real-time response desired (<1 h desirable)
**Assay protocol**	No reagent addition needed
**Operator**	Should be automated and require minimal operator skills
**Strain selectivity**	Ability to distinguish an individual bacterial strain from other strains of the same species
**Low detection limit**	Ability to detect single bacteria in a reasonably small sample volume (from 1 to100 mL)
**Species selectivity**	Ability to distinguish individual bacterial species in the presence of other microorganisms or cell.
**Compatible interface**	Should be compatible with the transduction principle and resist nonspecific binding
**Robustness**	Both mechanical and chemical stability is required
**Monitoring**	Direct, without pre-enrichment
**Viable cell count**	Should discriminate between live and dead cells

**Table 2:** Table based on phage-based biosensors in diagnosis of pathogens according to the type of phage, type of sample, duration of diagnosis and limit of detection achieved. Keys, NS, not stated.

Transducer	Organism	Bio-Probe Phage	Sample Matrix	Limit of Detection (CFU/mL)	Time Assay (min)	Ref
**Optical biosensor**	E. coli K12	T4	Skim milk	7 × 10^2^	NS	[3,56]
**Optical biosensor**	E. coli O157:H7	T4	Skim milk	3 × 10^3^	NS	[52,56,57]
**Optical biosensor**	MRSA	BP14	−	10^3^	NS	[52]
**Optical biosensor**	Salmonella	P22, TSP	Chicken carcass, wash	4.4 × 10^4^	3	[24,58,59]
**Optical biosensor**	C. jejuni	NCTC *12673 TSP*	−Contaminant milk	10^2^	45	[60,61]
**Bioluminesence**	E. coli	E. coli	−	Fewer than 10^3^	60	[62]
**Bioluminesence**	Salmonella enteritidis	SJ2	−	10^3^	120	[63]
**Fluorescent**	E.coli O157:H7	T7	Culture medium, water	10^7^	10	[40,64]
**QCM**	Salmonella typhimurium	Filamentous	Chicken wash	10^2^	60	[65,66]
**Impedimetric**	E. coli	T4	−	10^4^	30	[61,62,63]

**Table 3:** The nanoparticle phage-based biosensors in the diagnosis of pathogens according to type of phage, type of sample, duration of diagnosis and limit of detection.

Immobilization Material	Biosensor	Bio-Probe Phage	Organism	Limit of Detection (CFU/mL)	Ref
**Gold**	Fluorescence	T4	*E. coli*	ND	[73]
**Nano-aluminium fiber based filter**	Bioluminescence	Wild type and modified T4	*E. coli*	6 × 10^3^	[74]
**Silica particle**	Bioluminescence *lux* system	Phage A1122 with lux tag	*Yersinia pestis*	10^2^	[75]
**Carbon solid-phase extraction microarray**	Impediametric	T4	*E. coli* K12	10^2^–10^8^	[76]
**Carbon solid-phase extraction with magnetic beads**	Impediametric	T4	*E. coli* K12	10^2^–10^8^	[77]
**Pencil graphit electrodes with gold nano rods**	Impediametric	T4	*E. coli* K12	10^2^–10^6^	[78]

## Abbreviations

AK	adenylate kinase
ALP	alkaline phosphatase
CDC	centers for disease control and prevention
CFU	colony forming unit; ELISA, enzyme-linked immunosorbent assay
EIS	electrochemical impedance spectroscopy
HRP	horseradish peroxidase
LED	light emitting diode
MCLW	metal clad leaky waveguide
MRSA	methicillin-resistant *staphylococcus aureus*
Nluc	NanoLuc
PDPs	phage-display peptides
PCR	polymerase chain reaction
QCM	quartz crystal microbalance
RBPs	receptor binding proteins
R_ct_	resistance charge transfer
RT-PCR	reverse transcriptase PCR
RU	response unit
R&D	research and development
SPR	surface plasmon resonance
SEB	staphylococcal enterotoxin B
SPE	screen-printed graphene electrode
TE	transverse electric
TM	transverse magnetic
VBNC	viable-but non-cultivable
WHO	World Health Organization
